# Evaluating synergistic effects of metformin and simvastatin on ovarian cancer cells

**DOI:** 10.1371/journal.pone.0298127

**Published:** 2024-03-15

**Authors:** Sara Mikhael, Abdullah Kurdi, Nathalie Khoueiry-Zgheib, Roula Tahtouh, Rihab Nasr, George Hilal

**Affiliations:** 1 Laboratory of Cancer and Metabolism, Faculty of Medicine, Saint-Joseph University, Beirut, Lebanon; 2 Department of Biochemistry and Molecular Genetics, Faculty of Medicine, American University of Beirut, Beirut, Lebanon; 3 Department of Pharmacology and Toxicology, Faculty of Medicine, American University of Beirut, Beirut, Lebanon; 4 Department of Anatomy, Cell Biology, and Physiological Sciences, Faculty of Medicine, American University of Beirut, Beirut, Lebanon; National Institute of Cancer Research, TAIWAN

## Abstract

**Background:**

Ovarian Cancer (OC) stands as the most lethal gynecological malignancy, presenting an urgent clinical challenge in the quest to improve response rates. One approach to address this challenge is through drug repurposing, exemplified by the investigation of metabolic-modulating drugs such as Metformin (MTF) and Simvastatin (SIM). This study aims to explore the molecular mechanisms contributing to the potential synergistic anti-cancer effects between MTF and SIM on ovarian cancer cells.

**Methods:**

We assessed the effects of the combination on the proliferation and viability of two cell lines OVCAR-3 and SKOV-3. IC50 concentrations of MTF and SIM were determined using a proliferation assay, followed by subtoxic concentrations to explore the potential synergistic effects on the viability of both cell lines. Transcriptomic analysis was conducted on OVCAR-3 treated cells, and the findings were validated by assessing the expression levels of differentially expressed genes (DEGs) through real-time PCR in both cell lines SK-OV-3 and OVCAR-3.

**Results:**

Cytotoxicity analysis guided the selection of treatment concentrations as such MTF 10 mM and SIM 5 μM. The combined treatment of MTF and SIM demonstrated a synergistic inhibition of proliferation and viability in both cell lines. In OVCAR-3, exclusive identification of 507 DEGs was seen in the combination arm. Upregulation of FOXO3, RhoA, and TNFα, along with downregulation of PIK3R1, SKP2, and ATP6V1D levels, was observed in OVCAR-3 treated cells. Real-time PCR validation confirmed the consistency of expression levels for the mentioned DEGs.

**Conclusion:**

Our data strongly supports the presence of synergy between MTF and SIM in OC cells. The combination’s effect is associated with the dysregulation of genes in the key regulators AMPK and mTOR alongside other interconnected pathways.

## Introduction

Ovarian Cancer (OC) stands as the most lethal gynecological malignancy and ranks among the top five death-causing cancers in women worldwide [1, 2]. OC treatment includes surgery (oophorectomy) for staging and debulking with consideration given to combining intraperitoneal and intravenous adjuvant chemotherapy in advanced stages. The 5-year overall survival (OS) is as low as 25% in those stages because of delayed diagnosis, non-specific signs and symptoms, lack of appropriate screening tests, and resistance to standard platinum-based chemotherapy [2, 3]. Therefore, improving response rates in women diagnosed with OC is an urgent clinical issue.

Research into drug repurposing, an approach that explores novel therapeutic strategies, is gaining traction. This method involves using previously approved drugs with known pharmacokinetic and pharmacodynamic characteristics for indications other than their traditional ones, particularly in cancer treatment. Understanding the cellular features of tumors is important for drug repositioning with cellular metabolic reprogramming being one of the most prominent cancer hallmarks [4]. Metformin (MTF), a biguanide widely used for type II diabetes, targets these metabolic changes, leading to apoptosis and inhibition of cellular proliferation [5]. MTF has demonstrated its ability to restore metabolic homeostasis aberrantly reprogrammed in cancer cells. The anti-cancer effects of MTF have been well studied and reviewed in several *in vitro* and *in vivo* studies on various types of cancers, including breast, colorectal, small/non-small cell lung, head and neck, endometrial, and prostate cancers [4].

Statins, widely used for hypercholesterolemia and cardiovascular disease prevention, are also under study in cancer research. Statins competitively inhibit 3-Hydroxy-3-Methyl-Glutaryl-Coenzyme A (HMG-CoA) reductase crucial for the biosynthesis of cholesterol and several non-steroidal isoprenoid derivatives. Research has demonstrated that statins’ anti-cancer properties arise from inducing apoptosis, suppressing tumor growth, migration, invasion, and angiogenesis [6].

The combination of metabolic-modulating medications, MTF and SIM, was investigated in a preclinical study involving both *in vitro* assays and *in vivo* models in prostate cancer. The data showed a significant and synergistic reduction in cell viability and metastatic properties with lower toxicity observed in non-cancerous prostate epithelial cells. Subsequent evaluation in an orthotopic mouse model highlighted the combination’s significant inhibitory effects on primary tumor development, metastasis, and chemoresistance [7]. However, researchers have not yet explored this combination in OC in preclinical or clinical trials. While a retrospective cohort study hints at an association of better survival for ovarian cancer patients using metabolic targeting medications, conclusive findings are lacking [8].

Therefore, this *in vitro* study aimed to assess the anti-cancer effects of combining MTF and SIM on ovarian cancer cells, potentially offering a novel perspective for OC treatment in addressing the urgent need for improved response rates.

## Materials and methods

### Cell culture

In this study, two ovarian cancer cell lines, namely OVCAR-3 and SK-OV-3, were used. OVCAR-3 cells, isolated in 1982 from the malignant ascites of a patient with progressive adenocarcinoma of the ovary, were obtained from American Type Culture Collection (ATCC, Manassas, VA, USA). SK-OV-3 is an epithelial morphology cell line derived from the ovary of a 64-year-old, White female with ovarian adenocarcinoma. Both were maintained in the laboratory using Dulbecco’s Modified Eagle Medium/Nutrient mixture F-12 (DMEM/F12) (Sigma-Aldrich, Munich, Germany), supplemented with 20% Fetal Bovine Serum (FBS), and 1% penicillin-streptomycin (PS) (Sigma Chemical Co., St. Louis, MO, USA). Cells were incubated at 37°C in a humidified atmosphere containing 5% CO2.

### Chemicals

Metformin: 1,1-Dimethylbiguanide hydrochloride was obtained from Sigma-Aldrich in white solid form with a molecular weight of 165.62g/mol. A working concentration of 100mM was prepared. Simvastatin was obtained from Sigma-Aldrich in white solid form with a molecular weight of 418.57g/mol soluble in DMSO (≥20 mg/mL). The working concentration was diluted to 500μM prepared on the day of use, using a culture medium without FBS supplementation.

### Cell Proliferation analysis

Cytotoxic concentrations of the treatment conditions were determined using the colorimetric method based on the oxidation of 2-(2-methoxy-4-nitrophenyl)-3-(4-nitrophenyl)-5-(2, 4-disulfophenyl)-2H-tetrazolium and monosodium salt (WST-1) (Sigma-Aldrich, Munich, Germany) in three independent experiments (n = 3). OVCAR-3 cells and SK-OV-3 were plated and grown in a 96-well plate at a concentration of 6 x 10³ cells/well after performing a growth curve with different cell numbers. The cells were subsequently treated with varying concentrations of MTF (0-100mM) and SIM (0–100μM) as monotherapy at three time points: 24, 48, and 72h. 10μL of WST1 was added after 24, 48, and 72 hours respectively to each plate. The absorbance of the samples was measured using a Multiscan Go spectrophotometer (Thermo Scientific) at 450 nm every 15 min. Data were plotted as the mean ± SEM using GraphPad Prism.

### Cell Viability and synergy analysis

Trypan blue staining was performed on OVCAR-3 and SK-OV-3 to assess the synergistic effects between MTF and SIM on cellular proliferation and viability that will be expressed as percentage growth relative to control and to determine the quantitative synergism of the drugs when combined. The relative viability was determined when the cells were exposed to MTF and SIM alone and compared with the combination. 5 ⨯ 10⁴ cells/well were seeded in a 24-well plate 24 hours before treatment with 5 concentrations of MTF (0-40mM) and SIM (0–20μM) as a single treatment or in combination. According to Chou and Talalay’s method, synergy analysis was performed using CompuSyn software. Based on Chou’s experimental design for combination studies, a set of five data points (concentrations) should be selected as such ¼, ½, 1, 2, and 4 of the desired concentration (IC30 in this study). MTF concentrations include 2.5, 5, 10, 20, 40mM and SIM concentrations include 1.25, 2.5, 5, 10, 20ɥM. These effects were tested in three independent experiments. Fraction affected (Fa), that is, the fraction of cells inhibited by the drug, were determined. Briefly, synergism, additivity, or antagonism in the different combinations was calculated using the combination index (CI), where CI<1 indicates synergism, CI = 1 indicates additive effect, and CI >1 indicates antagonism [9].

### RNA isolation, purification, and quality assessment

RNA was isolated and purified from three independent experiments (n = 3) using Nucleozol reagent according to the manufacturer’s protocol (MACHEREY-NAGEL GmbH & Co. KG Düren, Germany). RNA quality was evaluated using the A260/A280 (1.7 to 2.1) and A260/230 (>1.7) ratios using a NanoDrop^®^ spectrophotometer. Denaturing 1% agarose gel electrophoresis was performed to assess RNA integrity.

### Gene expression microarrays

#### Conditions

Three independent experiments of OVCAR-3 cells were used for the gene expression microarrays. 1.5 x 10⁶ cells were seeded in Petri dishes 24 hours before treatment. Concentrations were as such: SIM 5μM and MTF 10mM.

#### Manual target preparation for GeneChipTM Whole Transcript (WT) expression arrays

Transcriptome profiling was performed using an Affymetrix GeneChip^™^ WT PLUS Reagent Kit. The GeneChip^™^ WT PLUS Reagent Kit used for transcriptome analysis was purchased from Thermo Fisher (Thermo Fisher Scientific) (Waltham, MA, USA). All samples were diluted to reach a starting point of 100ng of total RNA, which was reverse transcribed. For the reverse transcription, total RNA was primed with primers containing a T7 promoter sequence (Affymetrix WT cDNA Synthesis and Amplification Kit). The reaction synthesized single-stranded cDNA with a T7 promoter sequence at the 5’ end. The latter was converted to double-stranded cDNA, which was used as a template for in vitro transcription. DNA polymerase and RNase H were used to simultaneously degrade the RNA and synthesize second-strand cDNA. Antisense RNA (complementary RNA or cRNA) was synthesized and amplified by in vitro transcription (IVT) of the second-stranded cDNA template using T7 RNA polymerase. The enzymes, salts, inorganic phosphates, and unincorporated nucleotides were removed to prepare cRNA for 2nd-cycle single-stranded cDNA synthesis (ss-cDNA). RNase H was used to hydrolyze the cRNA template, leaving a single-stranded cDNA. After hydrolysis, the 2nd-cycle single-stranded cDNA (ss-cDNA) was purified to remove the enzymes, salts, and unincorporated dNTPs. This step prepped the cDNA for fragmentation and labeling. The purified sense-strand cDNA was fragmented using uracil-DNA glycosylase (UDG) and apurinic/apyrimidinic endonuclease 1 (APE 1). The fragmented cDNA was labeled with terminal deoxynucleotidyl transferase (TdT) using the proprietary DNA Labeling Reagent, which is covalently linked to biotin. Fragmented and labeled cDNA was hybridized to the Clariom^™^ S Human Transcriptome Array (Affymetrix, Inc., Santa Clara, CA, USA) at 45 °C for 17 h using the GeneChip^™^ 645 hybridization oven. The arrays were washed, stained on the FS450 Fluidics Station, and scanned in a GeneChip Scanner 3000 7G (Affymetrix, Inc., Santa Clara, CA, USA) according to the GeneChip^™^ User Guide.

#### Data analysis

Raw Affymetrix microarray images CEL files were normalized and transformed using Guanine Cytosine Counts Normalization (GCCN) and Signal Space Transformation (SST), respectively, by applying the apt-cel-transformer command from Affymetrix Power Tools (APT) version 1.20.0. The corrected CEL files were imported into R version 4.1.1 using read. celfiles function from the oligo Bioconductor R package version 1.58.0, with a data frame of the samples and the treatments used for each triplicate. Subsequently, the imported samples were subjected to quality checks using the arrayQualityMetrics function from the arrayQualityMetrics Bioconductor package version 3.50.0. After inspection of the samples using the generated report from the previous step, the samples were subjected to background adjustment to account for the noise in the optical detection system, normalization across arrays to compare measurements due to some sources of variation, such as laboratory conditions and batch effects, and summarization using the RMA algorithm to summarize multiple probes intensities for each gene into one quantity. These three steps were performed using the RMA function of the oligo package. Differentially expressed genes for MTF versus control, SIM versus control, and Combination versus control were identified by fitting the normalized and corrected matrix into a linear model. The LmFit function from the limma Bioconductor package version 3.50.0 was used to fit the data into a linear model followed by empirical Bayes correction using the eBayes function from limma. Differentially expressed genes with a Fold Change of 2 and p-value < 0.05 were kept for analysis. The transcripts were annotated using the annotateEset function from affycoretools Bioconductor package version 1.66.0 using pd.clariom.s.human Affymetrix annotation version 3.14.1 provided by Bioconductor. Venn diagrams, volcano plots, and heatmap of the dysregulated genes from the three comparisons were plotted using the Venn diagram, ggplot2, and ComplexHeatmap R packages, respectively. The inclusion criteria consisted of differentially expressed genes with absolute log₂fold change│log₂FC│ ≥ 1 and p-value < 0.05.

#### Pathway analysis

PathfindR Bioconductor package version 1.6.3 [10] was used to identify enriched pathways and enriched gene ontology terms (GO) for the uniquely dysregulated genes in combination vs control (507 genes), using the Kyoto Encyclopedia of Genes and Genomes (KEGG) pathways database and Gene Ontology (GO) Biological Process. KEGG pathways aid in visualizing the involvement of DEGs in biological and signaling pathways. GO is a computational representation of the functions of enriched genes. The top 15 enriched pathways and gene ontology terms in the combination arm were represented using an enrichment plot.

### Gene expression by RT-PCR

To confirm the transcriptome analysis data, the expression of the selected DEGs was assessed in OVCAR-3 and SK-OV-3. cDNA was synthesized using an iScript cDNA Synthesis Kit (Bio-Rad, USA), from 1ug of RNA. 5x iScript reaction mix reagent along with the reverse transcriptase was added to the total RNA, then the mixture was incubated at 25ᵒC for 5 minutes, at 42ᵒC for 30 minutes, and finally 5 minutes at 85ᵒC. The SYBR Green PCR Kit (Bio-Rad) was used to amplify the cDNA samples. The primer sequences are available in the Supplementary Data (S1 Table). The qPCR cycler, CFX connect, was used to amplify the cDNA using a PCR program of 35 cycles. The 2^-ΔΔCt^ method was used to calculate the relative fold change in gene expression levels. All expression levels of DEGs were normalized to the expression of GAPDH that was used as an internal control.

### Statistical analysis

Statistical analysis of the results of the proliferation studies and qPCR was performed using GraphPad Prism software. Repeated measures, one-way analysis of variance (ANOVA) with post hoc Tukey Honestly significant difference (HSD) was applied to determine the significant differences between the different conditions. The experimental results are expressed as mean ± SEM. Differences between treated and untreated conditions were considered statistically significant at *p < 0.05, **p < 0.01, and ****p < 0.0001.

## Results

### Proliferation effects of MTF and SIM on ovarian cancer cells

**Fig 1:**
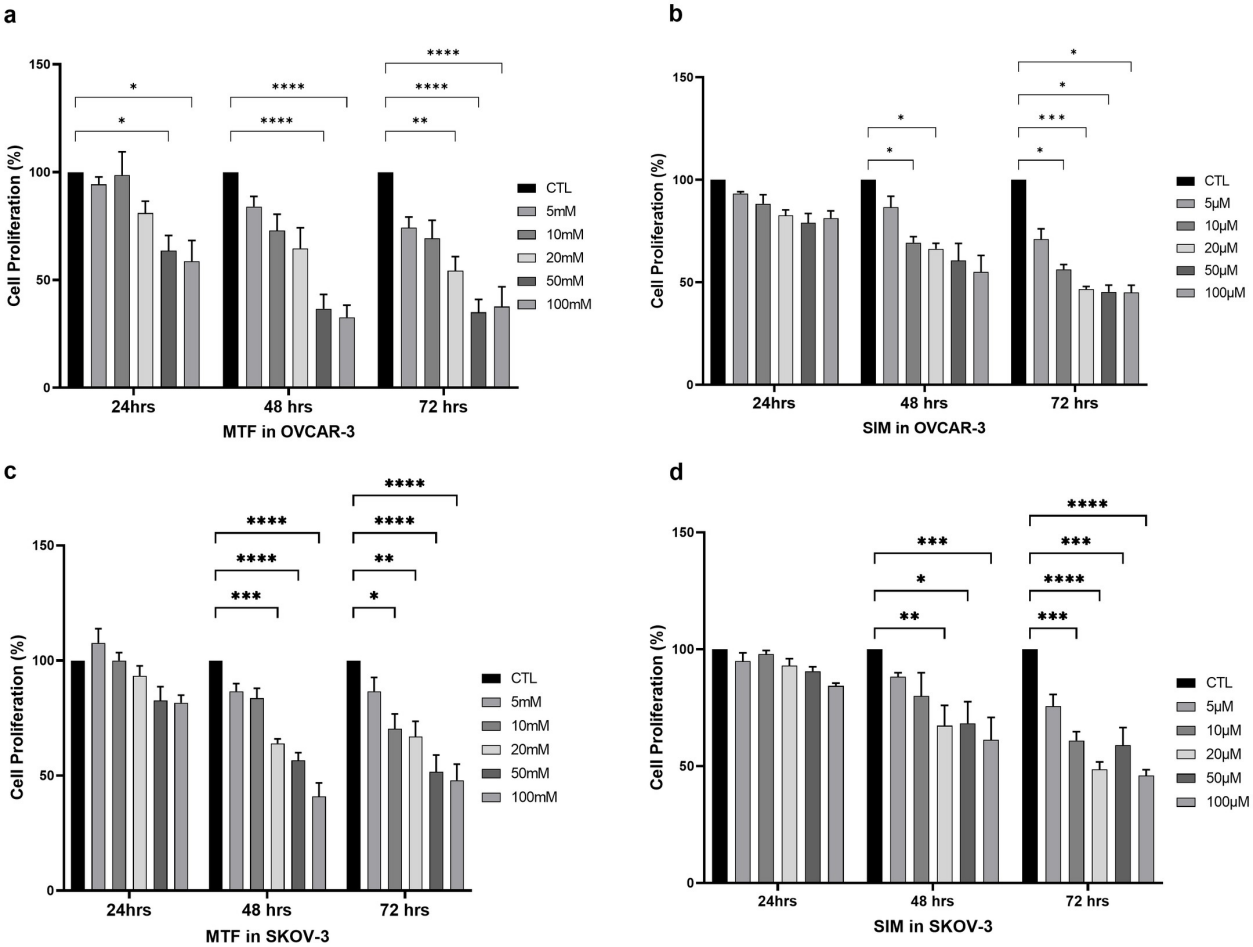
Proliferation effects. Dose-response curves of **(a)** Metformin [MTF] **(b)** Simvastatin [SIM] in OVCAR-3 cells. And (c) MTF and (d) SIM in SKOV-3 cells. Both showed a decrease in proliferation in dose and time dependent manners. (*p<0.05 **p<0.01 ****p<0.0001).

The effects of MTF and SIM on cellular proliferation were examined using OVCAR-3 and SK-OV-3 cell lines. The cells were exposed to varying concentrations of MTF(0-100mM) and SIM (0–100μM). The water-soluble tetrazolium salt (WST1) assay showed that both MTF and SIM, as monotherapies, decreased cell proliferation in a time and dose-dependent manner (Fig 1).

**Fig 2:**
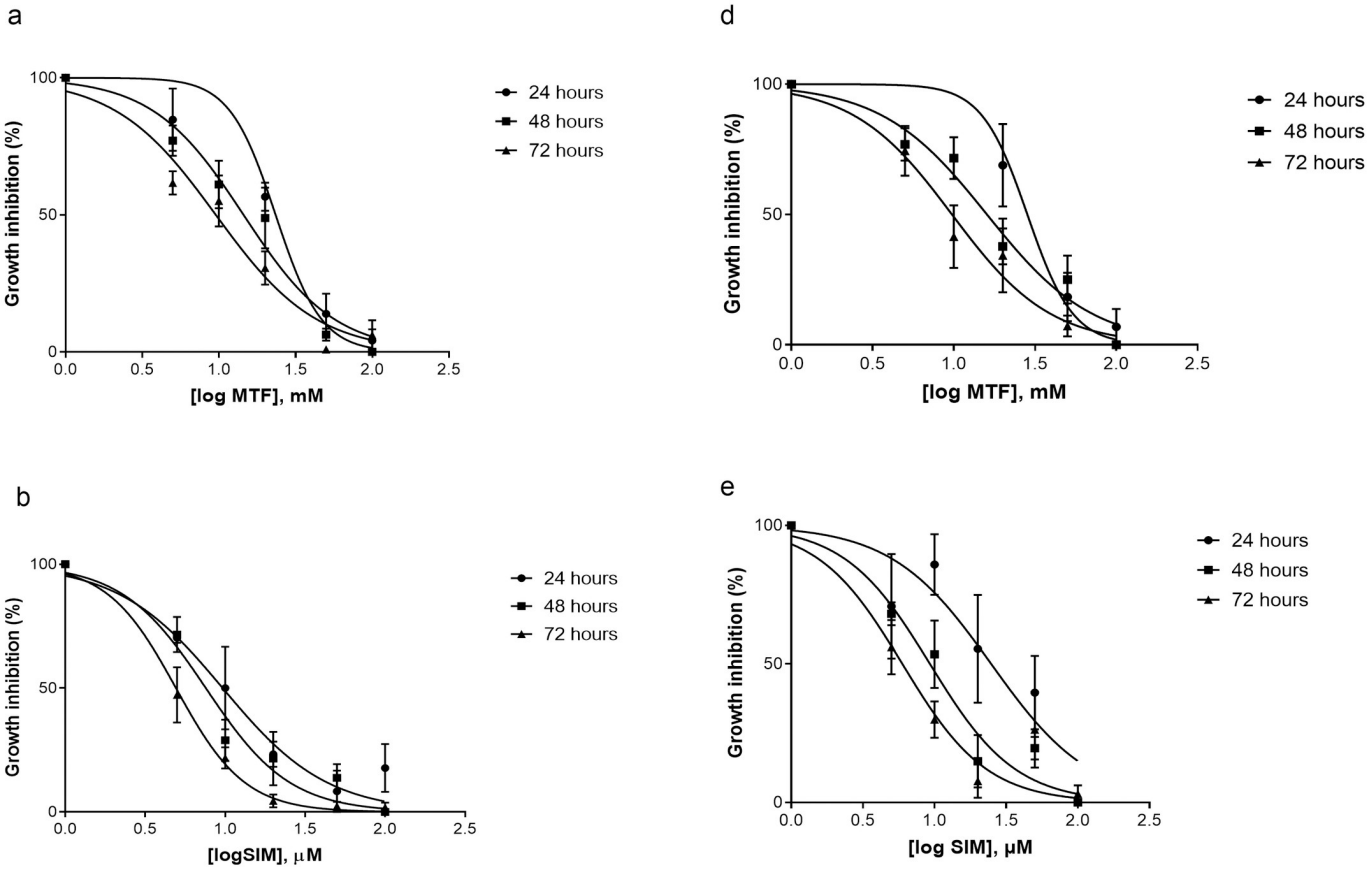
Cytotoxicity analysis and IC50 values. Dose-response curves of **(a)** Metformin [MTF] **(b)** Simvastatin [SIM] in OVCAR-3 at 24, 48, and 72 hours. MTF showed logIC50 values of 1.37mM, 1.16 mM, and 0.9 mM after 24, 48 and 72 hours respectively. SIM showed logIC50 values of 0.98μM, 0.88μM, and 0.69μM after 24, 48, and 72 hours respectively. **(c)** Metformin [MTF] **(d)** Simvastatin [SIM] in SK-OV-3 at 24, 48, and 72 hours. MTF showed logIC50 values of 1.45mM, 1.2 mM, 0.98 mM after 24, 48 and 72 hours respectively. SIM showed logIC50 values of 1.395μM, 0.96μM, and 0.77μM after 24, 48 and 72 h respectively. Dose-response curves are p2lotted as normalized mean SEM using GraphPad Prism (n = 3).

In OVCAR-3, the calculated half-maximal inhibitory concentrations (IC50) of MTF showed logIC50 values of 1.37mM (IC50 = 23mM), 1.16 mM (IC50 = 14.48mM), 0.9 mM (IC50 = 9.5mM), after 24, 48 and 72 hours respectively (Fig 2a). SIM showed logIC50 values of 0.98μM (IC50 = 9μM), 0.88μM (IC50 = 7μM), and 0.69μM (IC50 = 5μM) after 24, 48 and 72 h respectively (Fig 2b). In SK-OV-3, the calculated half-maximal inhibitory concentrations (IC50) of MTF showed logIC50 values of 1.45mM (IC50 = 28mM), 1.2 mM (IC50 = 15.99mM), 0.98 mM (IC50 = 9.7mM) after 24, 48 and 72 hours (Fig 2c). SIM showed logIC50 values of 1.395μM (IC50 = 25μM), 0.96μM (IC50 = 9μM), and 0.77μM (IC50 = 6μM) after 24, 48 and 72 h respectively (Fig 2d).

### Viability effects of MTF and SIM in ovarian cancer cells

**Fig 3:**
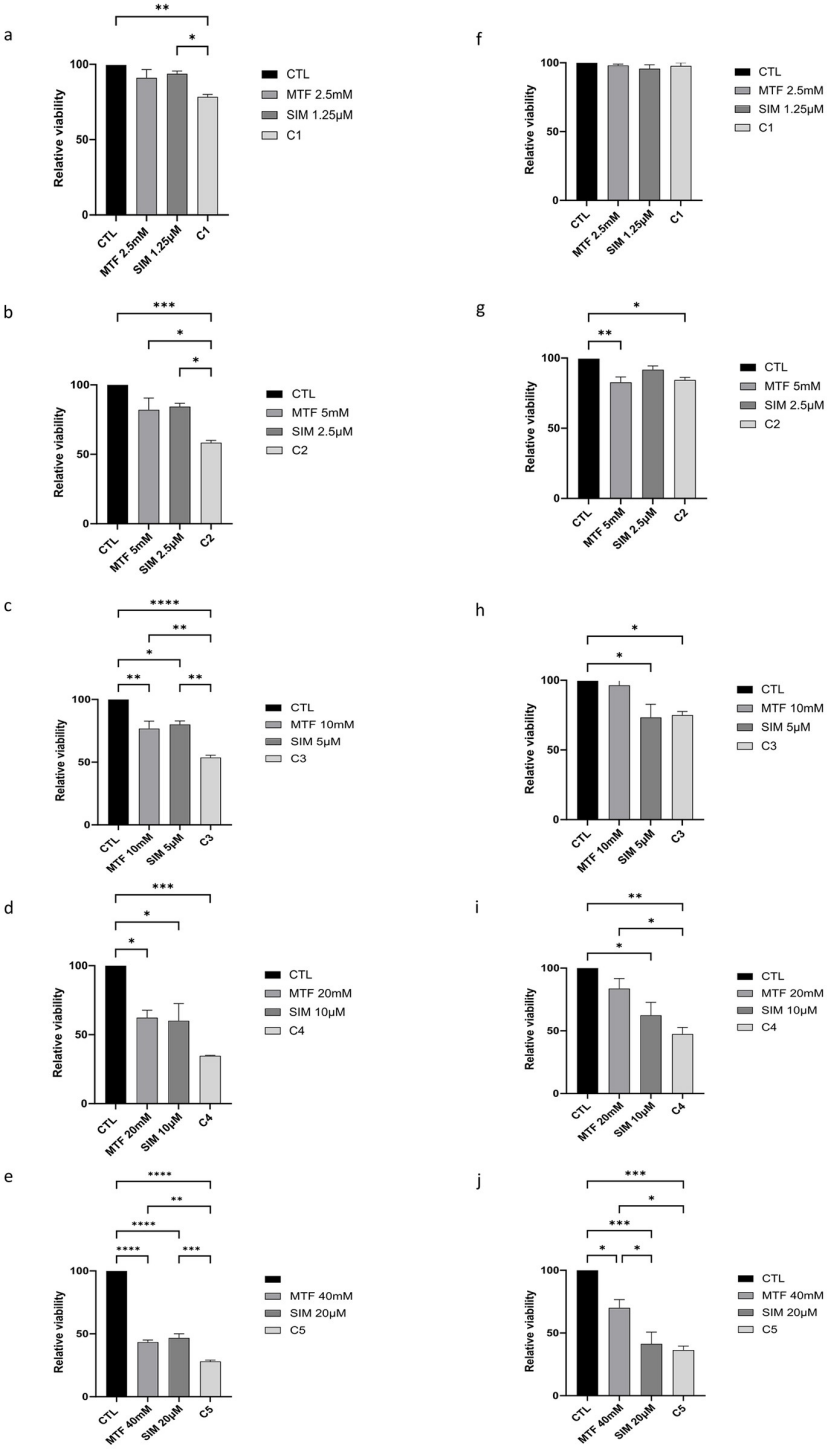
Proliferation and Viability effects. Studied by trypan blue count. (a-e) Three replicates of OVCAR-3 cells and (f-j) SKOV-3 cells were treated with increasing concentrations with single therapies of MTF 2.5mM, 5mM, 10mM, 20mM, 40mM) or SIM (1.25μM, 2.5 μM, 5 μM, 10 μM, and 20 μM) or their combination (MTF+SIM). Dose-response curves are plotted as normalized mean SEM using GraphPad Prism (n = 3) (*p<0.05 **p<0.01 ****p<0.0001).

We used IC30 concentrations to calculate the dose ranges for the trypan blue staining. The effects of MTF and SIM, alone or in combination, on the proliferation and viability of the OVCAR-3 and SKOV-3 cells, are shown in Fig 3. In OVCAR-3 and as shown in Fig 3a–3e, SIM as a monotherapy showed statistically significant effects at concentrations of 5μM (Fig 3c) and higher when compared with untreated control samples. MTF monotherapy resulted in a statistically significant decrease in the relative viability starting at concentrations of 10mM (Fig 3c) and higher when compared with untreated control samples. However, combining MTF and SIM significantly decreased cellular viability at all concentrations (Fig 3a) starting with the lowest C1 (MTF 2.5mM and SIM 2.5μM) with p = 0.0045; C2 (MTF 5mM and SIM 2.5μM) with p = 0.0008, C3 (MTF 10mM and SIM 5μM) with p<0.0001, C4 (MTF 20mM and SIM 10μM) with p = 0.0007, and C5 (MTF 40mM and SIM 20μM) with p<0.0001. As shown in Fig 3f–3j, when compared to untreated samples, MTF as a single therapy showed statistical significance effects on the viability and proliferation of SKOV-3 cells at the highest concentration 40mM (Fig 3j) with p = 0.03. SIM alone showed statistical significance effect on the proliferation and viability of SK-OV-3 cells when compared to the untreated control samples at concentrations of 5μM and higher (Fig 3h–3j). However, combining MTF and SIM showed significant decrease on the proliferation and viability of SKOV-3 cells at low concentrations such as C2 (MTF 5mM and SIM 2.5μM) with p = 0.0176, C3 (MTF 10mM and SIM 5μM) with p = 0.0399, C4 (MTF 20mM and SIM 10μM) with p = 0.0.34, and C5 (MTF 40mM and SIM 20μM) with p = 0.0003.

### Synergistic effects of combining MTF and SIM in ovarian cancer cells

**Fig 4:**
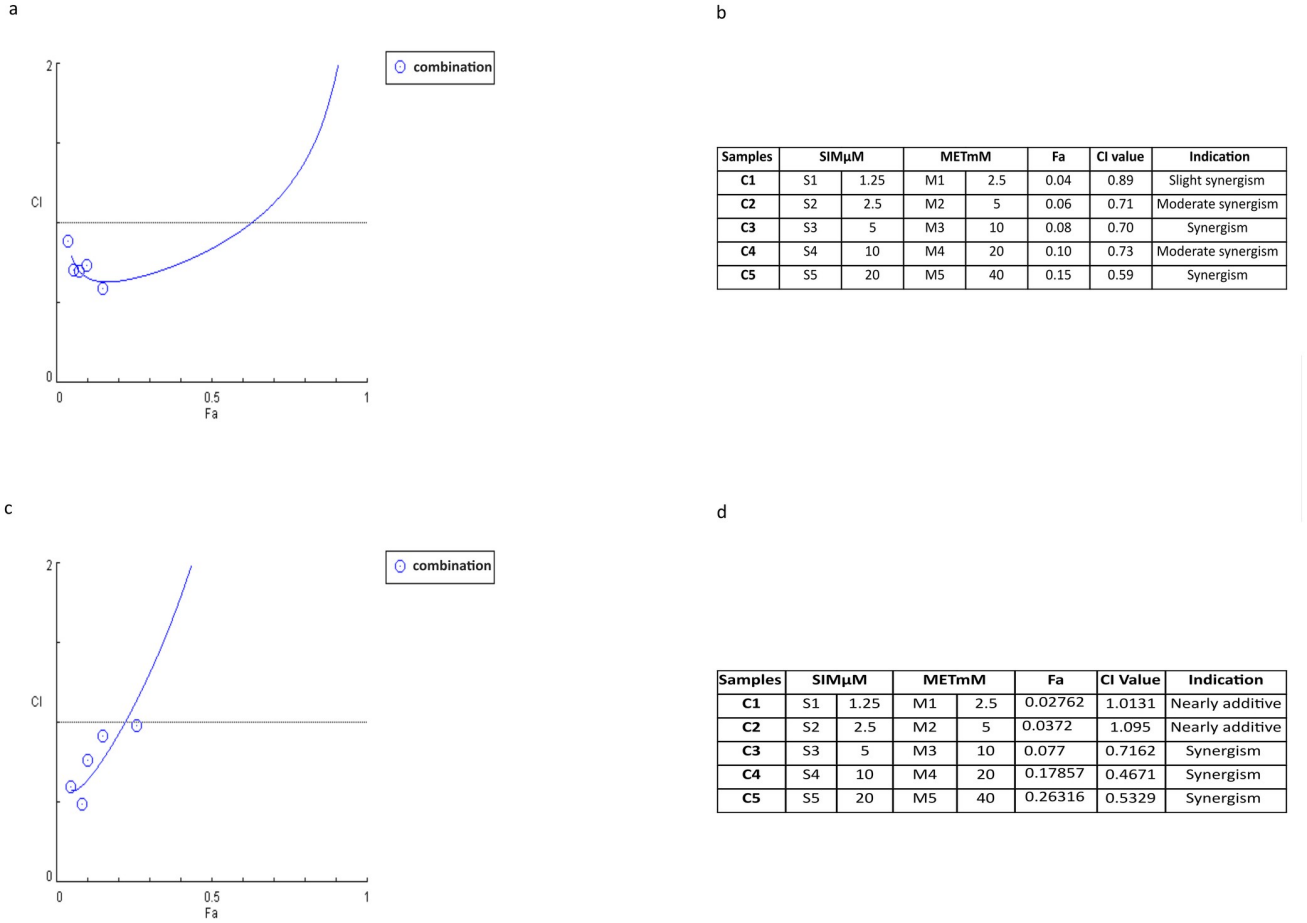
Synergy analysis. **(a)** Fa-CI plot illustration of one biological replicate of OVCAR-3 cell line with its **(b)** corresponding values. **(c)** Fa-CI plot illustration of one biological replicate of SK-OV cell line with its **(d)** corresponding values. The graphs were generated by the computerized software CompuSyn. The circles in the graph indicate the CI values. Tables b and d summarize the compuSyn results of the specific concentrations for each drug when combined with their relative Fa and CI values. CI<0.1 Very strong synergism, CI = 0.1–0.3 Strong synergism, CI = 0.3–0.7 Synergism CI = 0.7–0.85 Moderate synergism, CI = 0.85–0.90 Slight synergism, CI = 0.90–1.10 Nearly additive, CI = 1.10–1.20 Slight antagonism, CI = 1.20–1.45 Moderate antagonism, CI = 1.45–3.3 Antagonism, CI = 3.3–10 Strong antagonism, CI>10 Very strong antagonism. Fa: fraction affected, CI combination index.

The results in Fig 4 are based on the combination index (CI) method in which CI was calculated using CompuSyn [9]. In OVCAR-3 (Fig 4a and 4b) the combination of MTF and SIM showed slight to moderate synergism. The combination of 10mM MTF and 5μM SIM, with a calculated CI of 0.699, was selected for transcriptomic experiments due to its synergistic effects at the IC30 concentrations of MTF and SIM. In SK-OV-3 (Fig 4c and 4d) the combination of MTF and SIM showed additive effects in C1 and C2 while synergistic effects in the following concentrations C3, C4 and C5.

### Effects of MTF and SIM on OVCAR-3 cell transcriptome

**Fig 5:**
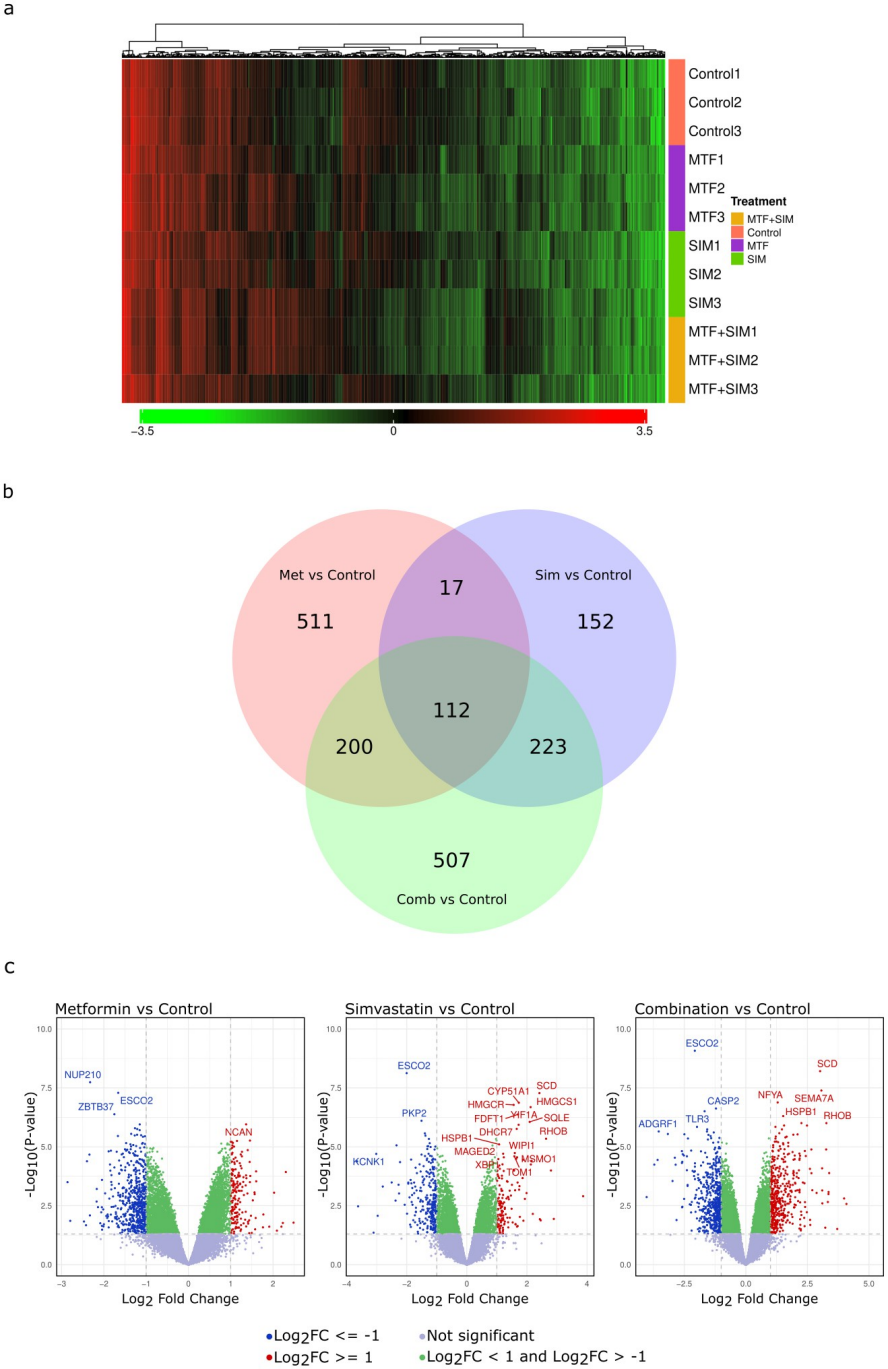
Transcriptome analysis data. (a) 2-dimensional heat map of normalized clustered intensities of the mRNA of the DEGs for the profiled samples for each condition; Log 2-Fold change │Log₂FC│≥ 2; P<0.05. (b) Venn diagram showing the number of DEGs whether exclusive or shared. 1722 in total. Log 2-Fold change │Log₂FC│≥ 1, P-value <0.05. (c) Volcano plots showing DEGs in each condition. Up-regulated genes are highlighted in red (Log2FC ≥ 1); Down-regulated genes are highlighted in blue (log2FC ≤ -1) Green highlighted genes have log2FC < 1 and > -1. All with a (p-value < 0.05). Insignificant genes with p-value> 0.05 are highlighted in grey.

To assess the impact of MTF and SIM on the transcriptome of OVCAR-3 cells, we subjected cells to treatments from three independent experiments (n = 3), administering 10 mM MTF and 5 μM SIM individually or in combination for a duration of 48 hours. RNA was isolated and purified using Nucleozol reagent. After performing manual target preparation for GeneChipTM Whole Transcript (WT) Expression Arrays, analysis was performed. Differentially expressed genes (DEGs) were visualized on a normalized 2-dimensional heat map of hierarchically clustered intensities of the mRNA of the DEGs for the profiled samples for each condition │log₂FC│ ≥ 2 (Fig 5a). The total number of DEGs was 1722 (S2 Table). 511 DEGs were exclusively found in the MTF vs. control group, 152 in the SIM vs. control, and 507 exclusives in the combination group. MTF and SIM groups shared 17 DEGs, MTF and the combination groups shared 200 DEGs and, SIM and the combination groups shared 223 DEGs. A total of 112 DEGs were identified in all groups (Fig 5b). 507 genes were shown to be exclusively regulated in the combination arm. Based on the p-value, the top upregulated DEG observed was SEMA7A (p-value: 4.61E-08; FC: 8.40) and the top downregulated DEG was PHACTR2 (p-value: 1.35E-05; FC: -2.46). Fig 5c shows three independent volcano plots showing the DEGs under each condition. Up-regulated genes are highlighted in red (Log2FC ≥ 1); down-regulated genes are highlighted in blue (log2FC ≤ -1), and green-highlighted genes have log2FC < 1 and > -1. Statistical significance was set to a p-value < 0.05. Insignificant genes with p-value> 0.05 are highlighted in grey.

### Pathways affected by the combination of MTF and SIM Using KEGG analysis and Gene Ontology

**Fig 6:**
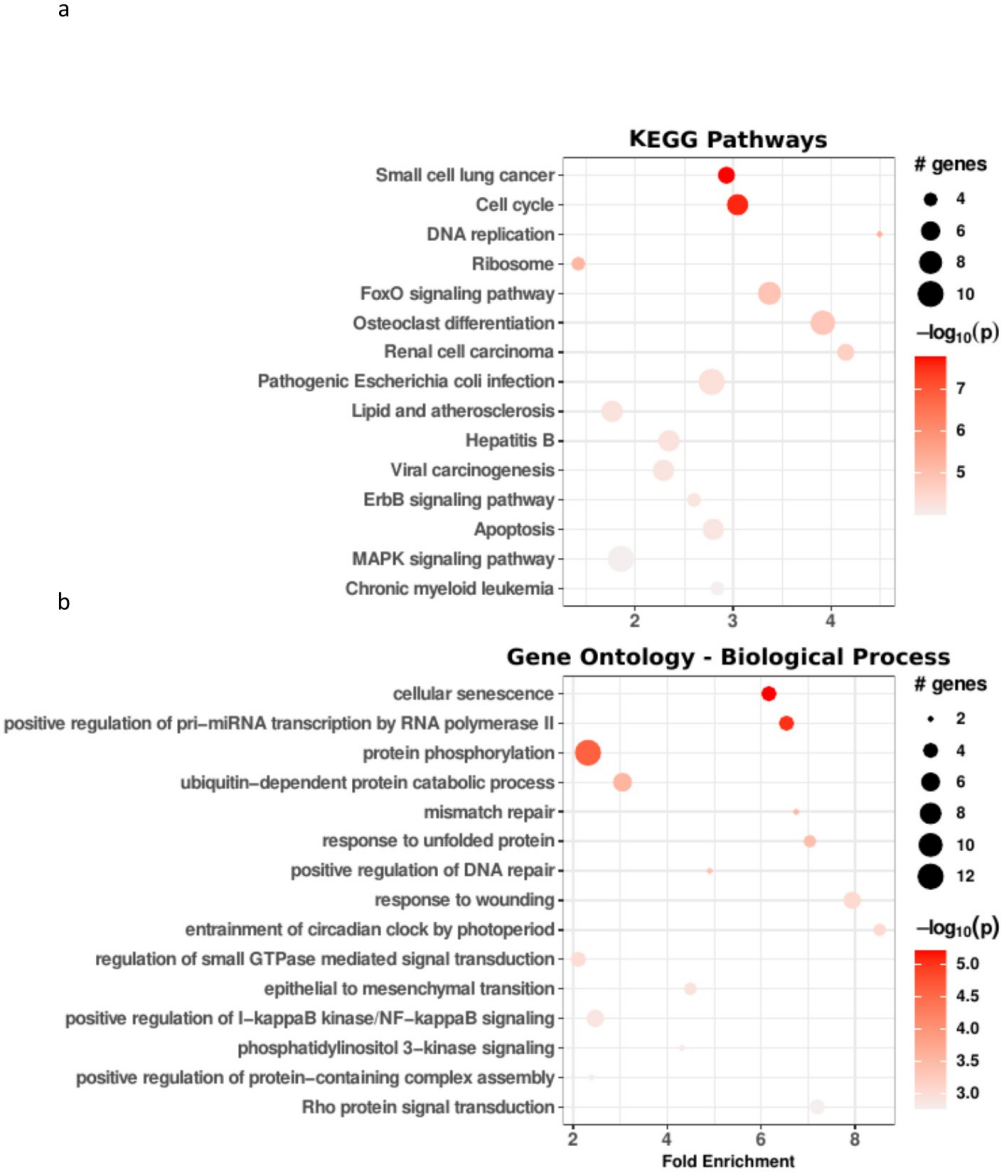
Pathway analysis and Gene ontology (GO). Enrichment analysis for the combination group exclusively shows **(a)** KEGG (Kyoto Encyclopedia of Genes and Genomes) pathways in the combination arm only. **(b)** GO (Gene ontology i.e biological process) The dots show the number of genes enriched in each pathway shown. The intensity of the red color in both analyses depends on the log fold change of enrichment.

To elucidate the biological characteristics of the synergistic combination, we used the DEGs found in the combination vs. control dataset for further downstream analysis. The pathways relevant to the effects of the combination in ovarian cancer included cell cycle, apoptosis, DNA replication, and MAPK signaling pathway (Fig 6a and 6b). We also examined the DEGs enriched in metabolic signaling pathways, specifically the AMPK and mTOR, which are known to be interconnected key regulators of cancer cell metabolism.

#### AMPK signaling pathway

Out of 507 genes, 3 were enriched in AMPK including 2 upregulated gene, FOXO3 (p-value: 0.0005; FC: 2.54), and PEPCK also named PCK2 (p-value: 0.049; FC: 2.87), and 1 downregulated gene: and PIK3R1 (p-value: 0.00255; FC: -2.087). KEGG analysis showed that AMPK was a direct activator of FOXO3. Another gene found to be differentially expressed in the exclusive combination dataset was PIK3R1. According to KEGG analysis, AMPK indirectly inhibits the PI3K-AKT signaling pathway by phosphorylating and activating TSC1/2. The third differentially expressed gene associated with AMPK is PCK2, also known as PEPCK-M (the mitochondrial isoform of Phosphoenolpyruvate carboxykinase). According to KEGG analysis, PCK2 is a component of the AMPK signaling pathway that is indirectly affected by AMPK through TORC2, and its expression was shown to be increased by the combination (S1a Fig).

#### mTOR signaling pathway

Of 507 genes, five were enriched in the mTOR pathway and included two upregulated genes: RhoA (p-value: 0.00011; FC: 2.12), TNFα (p-value: 0.00037; FC: 4.54), and three downregulated genes: PIK3R1(p-value: 0.00255; FC: -2.09), SKP2(p-value 0.0042; FC: -2.35), and ATP6V1D (p-value: 0.015; FC: -2.026). PIK3R1 is associated with the mTOR pathway. According to KEGG analysis, the insulin signaling pathway leads to the activation of PI3K, which in turn directly activates mTORC2 via Rictor phosphorylation and indirectly activates mTORC1 via AKT. Our data showed that the combination of MTF and SIM significantly decreased the expression of PIK3R1. Additionally, another indirect regulator of mTORC1 found in our analysis of the combined dataset is TNFα. Our data showed that MTF and SIM increased TNFα expression. MTF and SIM also synergistically decreased the expression of two key mTORC1 regulators, SKP2 and ATP6V1D (S1b Fig).

### Validation of DEGs by real-time PCR

**Fig 7:**
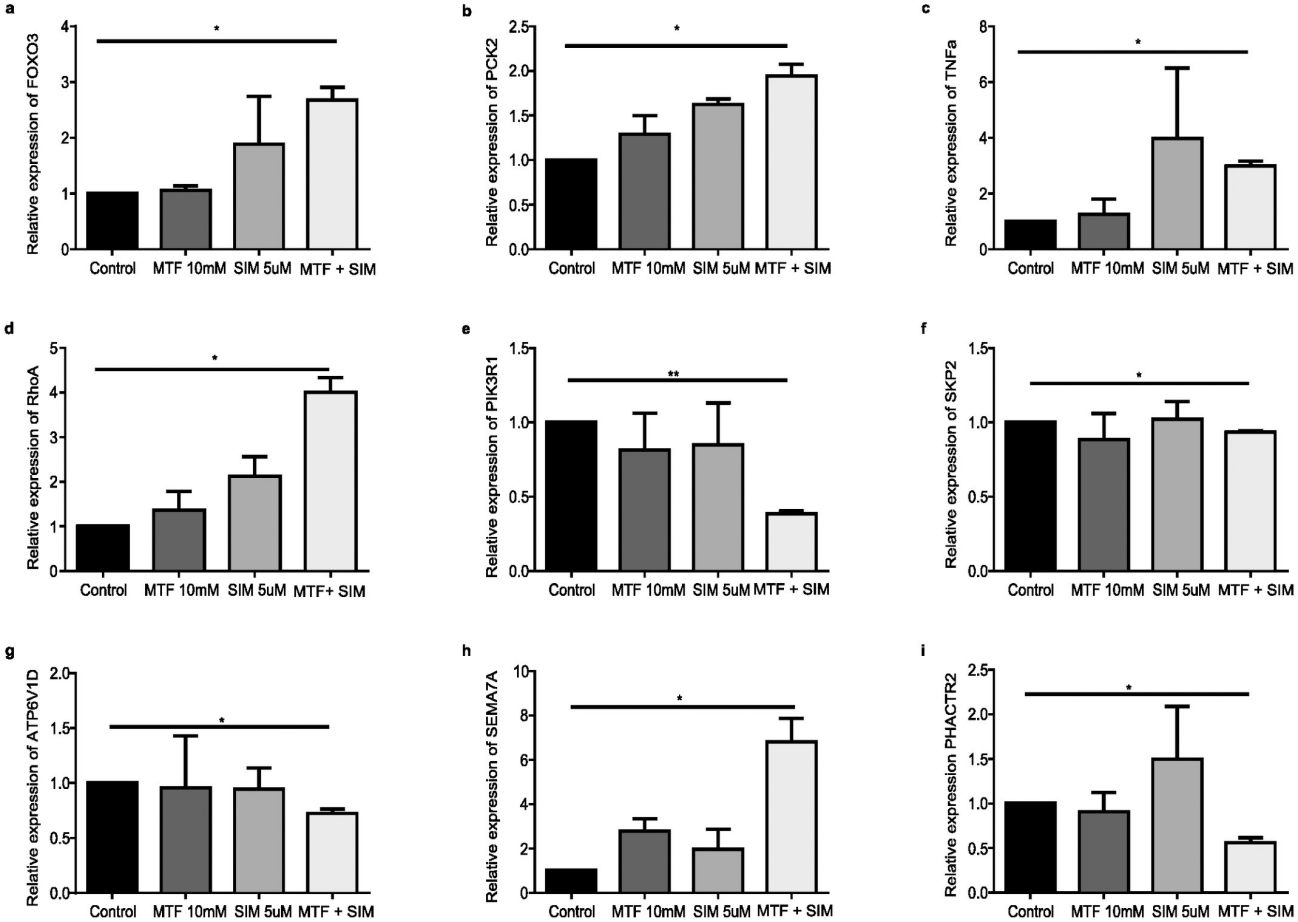
Validation of selected DEGs by real-time PCR in OVCAR-3. (a-g): Relative expression of the differentially expressed genes (DEGs) found to be enriched in AMPK and mTOR pathway. (h,i): Relative expression of the most up and downregulated genes respectively. Determined with Real time PCR Measurements with GAPDH as internal control. Treatment conditions were compared to untreated samples. Experiments were performed in triplicate. Graphs were plotted as mean SEM using graphpad Prism. (a) FOXO3 (ANOVA, Tukey’s HSD p = 0.0482), (b) PCK2 (ANOVA, Tukey’s HSD p = 0.0483), (c) TNFα (ANOVA, Tukey’s HSD p = 0.0229) and (d) RhoA (ANOVA, Tukey’s HSD p = 0.0302), (e) PIK3R1 (ANOVA, Tukey’s HSD p = 0.0016), (f) SKP2 (ANOVA, Tukey’s HSD p = 0.0423), and (g) ATP6V1D (ANOVA, Tukey’s HSD p = 0.0485), (h) SEMA7A (ANOVA, Tukey’s HSD p = 0.0367), (i) PHACTR2 (ANOVA, Tukey’s HSD p = 0.0380). Abbreviations: MTF: metformin; SIM: simvastatin; GAPDH: Glyceraldehyde 3-phosphate dehydrogenase, FOXO3: fockhead box O3; PCK2: Phosphoenolpyruvate Carboxykinase 2; TNFα: tumor necrosis factor alpha; RhoA: Ras homolog family member A; PIK3R1: Phosphoinositide-3-Kinase Regulatory Subunit 1; SKP2: S-phase kinase-associated protein 2; ATP6V1D: ATPase H+ Transporting V1 Subunit D; SEMA7A: Semaphorin 7A; PHACTR2: Phosphatase and Actin Regulator 2.

**Fig 8:**
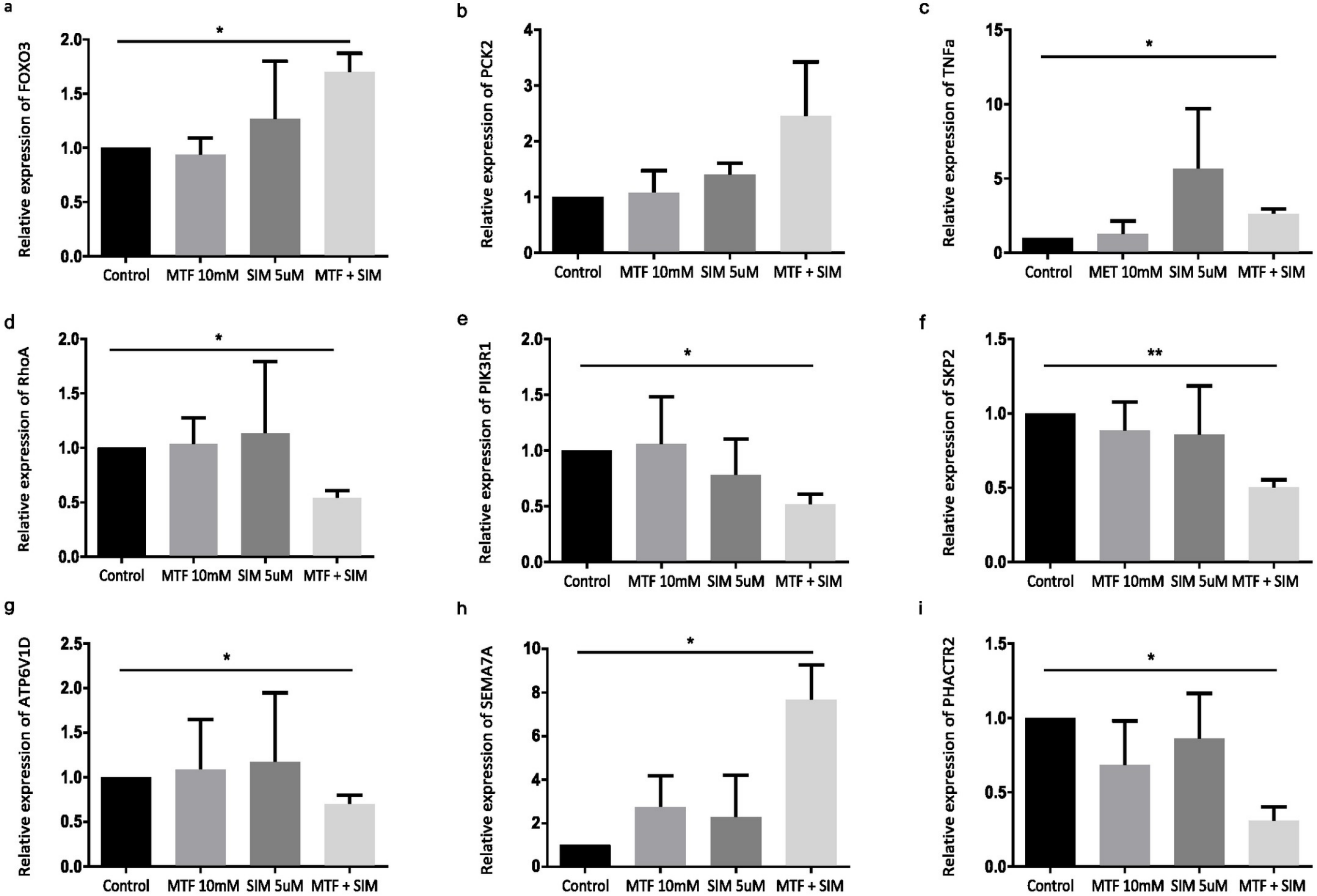
Validation of selected DEGs by real-time PCR in SK-OV-3. (a-g): Relative expression of the differentially expressed genes (DEGs) found to be enriched in AMPK and mTOR pathway. (h,i): Relative expression of the most up and downregulated genes respectively. Determined with Real time PCR Measurements with GAPDH as internal control. Treatment conditions were compared to untreated samples. Experiments were performed in triplicate. Graphs were plotted as mean SEM using graphpad Prism. (a) FOXO3 (ANOVA, Tukey’s HSD p = 0.049), (b) PCK2 (ANOVA, p>0.05), (c) TNFα (ANOVA, Tukey’s HSD p = 0.047), (d) RhoA (ANOVA, Tukey’s HSD p = 0.0156), (e) PIK3R1 (ANOVA, Tukey’s HSD p = 0.028), (f) SKP2 (ANOVA, Tukey’s HSD p = 0.0095), and (g) ATP6V1D (ANOVA, Tukey’s HSD p = 0.0467), (h) SEMA7A (ANOVA, Tukey’s HSD p = 0.046), (i) PHACTR2 (ANOVA, Tukey’s HSD p = 0.0156). Abbreviations: MTF: metformin; SIM: simvastatin; GAPDH: Glyceraldehyde 3-phosphate dehydrogenase, FOXO3: fockhead box O3; PCK2: Phosphoenolpyruvate Carboxykinase 2; TNFα: tumor necrosis factor alpha; RhoA: Ras homolog family member A; PIK3R1: Phosphoinositide-3-Kinase Regulatory Subunit 1; SKP2: S-phase kinase-associated protein 2; ATP6V1D: ATPase H+ Transporting V1 Subunit D; SEMA7A: Semaphorin 7A; PHACTR2: Phosphatase and Actin Regulator 2.

To ensure the validity of our transcriptomic analysis, we evaluated the expression levels of selected DEGs using real-time PCR on two cell lines OVCAR-3 and SK-OV-3. In OVCAR-3, results showed a statistically significant upregulation of FOXO3 (p = 0.0482), PCK2 (p = 0.0483), TNFα (p = 0.0229), and RhoA (p = 0.0302) (Fig 7a–7d), along with a statistically significant downregulation of PIK3R1 (p = 0.0016), SKP2 (p = 0.0423), and ATP6V1D (p = 0.0485) (Fig 7e–7g) in the combination group. The most up-regulated and down-regulated genes were also validated, namely SEMA7A (p = 0.0367), (Fig 7h), and PHACTR2 (p = 0.0380) (Fig 7i). All expression levels were consistent with the transcriptome profile OVCAR-3 cells in the combination group. SK-OV-3 data were similar to OVCAR-3 with a significant upregulation of FOXO3 (p = 0.049) and TNF (p = 0.047) (Fig 8a and 8c) along with a significant downregulation of PIK3R1 (p = 0.028), SKP2 (p = 0.0095), and ATP6V1D (p = 0.0467) (Fig 8e–8g) in the combination group. Additionally, the most up-regulated and down-regulated genes were also validated in SK-OV-3, namely SEMA7A (p = 0.046), (Fig 8h), and PHACTR2 (p = 0.0156) (Fig 8i). However, contrary to results found in OVCAR-3 the combination of MTF and SIM in SK-OV-3 did not yield a significant increase in PCK2 expression (p>0.05) (Fig 8b) and the combination decreased RhoA expression (p = 0.0157) (Fig 8d)

## Discussion

**Fig 9:**
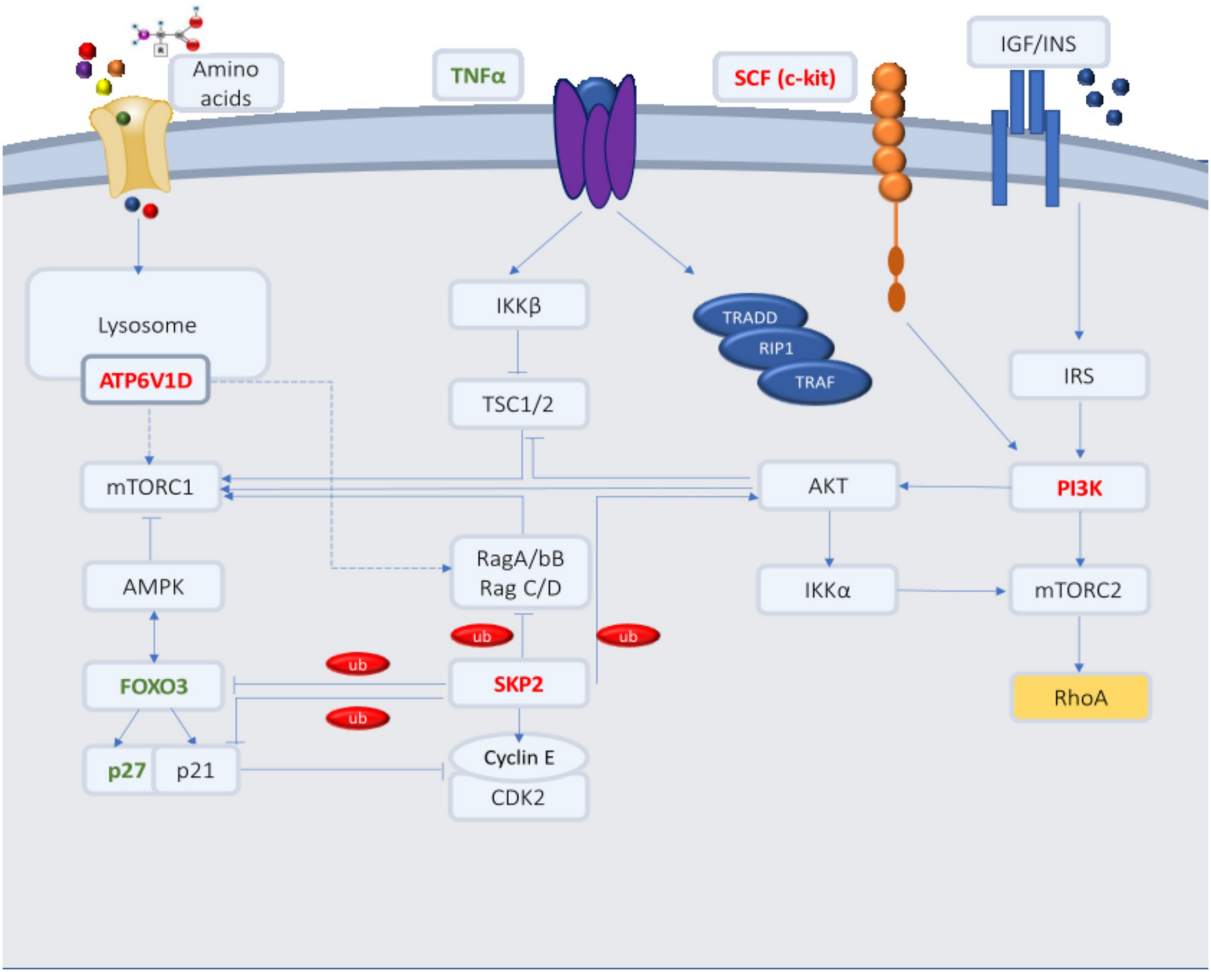
Crosstalk between mTOR and AMPK. mTOR (mammalian target of rapamycin) and AMPK (AMP-activated protein kinase) are found to be enriched in KEGG (Kyoto Encyclopedia of Genes and Genomes) analysis. Colored genes are found to be enriched in those pathways and are found to be regulated in the combination arm exclusively. Green highlighted genes are found to be upregulated by the combination. Red highlighted genes are found to be downregulated by the combination. Yellow highlighted gene (RhoA) was shown to be upregulated in OVCAR-3 but downregulated in SK-OV-3. DEGs enriched in AMPK include PI3K and FOXO3. According to KEGG, FOXO3 is a direct target of AMPK forming a positive feedback loop. DEGs enriched in mTOR signaling pathway include ATP6V1D, PI3K, SKP2, TNF that control mTOR signaling pathway through different trails. KEGG analysis shows that RhoA is a direct target of mTORC2. PI3K is activated by insulin receptor substrate 1 (IRS1), a signaling protein that is phosphorylated and activated upon the interaction between insulin growth factor and its receptor or even by SCF (c-kit ligand) that is also shown to be targeted by the combination of MTF and SIM. Activated PI3K activates mTORC2 by direct phosphorylation or by indirect activation via IKKα. Second it activates AKT which in turn activates mTORC1 directly or indirectly via inhibition of TSC2 or IKKα. SKP2 inhibits FOXO3, p21, p27, RagA/B, C/D and activates cyclin E, CDK2 and AKT. ATP6V1D activates RagA/B and Rag C/D; dotted arrow: indirect activation (Original figure).

In this study, we demonstrated the synergistic effects of combining MTF and SIM on the proliferation and viability of both OVCAR-3 and SK-OV-3 cell line. The two cell lines used in this study represent two distinct pathological and behavioral states, with OVCAR-3 being a high-grade serous ovarian carcinoma derived cell line (HGSOC), associated with a missense exon 7 tp53 mutation, late-stage diagnosis, and rapid peritoneal spread. On the other hand, SK-OV-3, the non-serous carcinoma cell line, derived from ascitic fluid, associated with endometriosis, is more commonly present at an early stage with a loss of function mutation (LOF) for tp53 that results in no p53 protein expression (p53 null) [11]. In addition to losing the tumor suppressive role, the absence or decreased function of p53 in cancer cells obtain new oncogenic features such as enhancing angiogenesis, invasion, migration, metabolic reprogramming and chemoresistance [11]. Despite HGSOC generally exhibiting a more aggressive clinical behavior, SK-OV-3 displayed greater aggressiveness in terms of proliferation and viability. This paradox may be attributed to SKOV-3’s origin from ascitic fluid, linking it to a more advanced disease [11, 12]. Our observation of lower IC50 values, higher viability, proliferation, and synergistic effects of both drugs on OVCAR-3 compared to SK-OV-3 aligns with existing studies. A study by Kobayashi et al. [13] showed that SKOV-3 possessed a more invasive capacity than OVCAR-3 cell line. Another study by Choi et al. [14] showed that SKOV-3 cells have a higher EGFR expression with a greater capacity to invade than OVCAR-3. Rogalska et al. [15] and Faramarzi et al. [16] both demonstrated that the IC50 of MTF when treating SKOV-3 cells was 14mM and 14.92mM respectively, similar to our data. Another study by Huo et al. [17] also showed that MTF (0-20mM) had an inhibitory effect on the proliferation of SKOV-3 cells Our study marks a significant contribution as the first to reveal the impact of combining MTF and SIM on the proliferation and viability of ovarian cancer cells. Notably, this combination demonstrated a synergistic growth inhibiting effect on endometrial and prostate cancer cell lines [18, 19]. Additionally, we performed the first transcriptome profiling of OVCAR-3 treated cells with this combination. We identified more than 500 DEGs when the two drugs were combined. Enrichment analysis showed that the combination targets genes involved in various critical pathways including cellular senescence, apoptosis, cell cycle, DNA replication, EMT, mismatch repair, lipolysis, insulin resistance, glycolysis, MAPK and other signaling pathways (Fig 4). From a metabolic perspective, this was significantly associated with the dysregulation of some of the components of the metabolic sensors, namely AMPK and mTOR as illustrated in Fig 9.

AMP-activated protein kinase (AMPK) functions as a crucial sensor for cellular metabolism playing a pivotal role in maintaining cellular energy homeostasis and regulating glucose, protein, and lipid metabolism [20]. Our study shows that the combination of MTF and SIM activates Forkhead box O3a (FOXO-3a), a key transcription factor in the AMPK network. This activation aligns with a study by Queiroz et al. [21], which demonstrated increased FOXO3a in breast cancer cell lines treated with 10mM of MTF. Activated FoxOs are pivotal tumor suppressors involved in the transcription of several genes that in turn regulate fundamental cellular processes such as apoptosis, cell cycle arrest, glucose metabolism, and DNA repair. A study by Fei et al. [22] showed that the expression of FoxO3a was highest in normal ovarian cells and lowest in malignant tumors. FoxO3a expression is also associated with disease prognosis where patients with higher FoxO3a expression have a higher overall survival rate. Another study by An et al. [23] showed that there is a positive feedback loop mechanism between AMPK and FOXO3a, in which AMPK phosphorylates and promotes the translocation of FoxO3a to the nucleus enabling its activation.

Our study also showed that the combination of MTF and SIM increased the expression of PCK2 (phosphoenolpyruvate carboxykinase 2) also known as PEPCK-M, the mitochondrial isoform of PEPCK- a key enzyme linked to gluconeogenesis [24]. Interestingly, conflicting data exist regarding its function in cancer. According to KEGG analysis, AMPK inhibits PCK2. However, a recent study by Xiong et al showed that PCK2 expression inhibited the progression of renal cell carcinoma (RCC) and enhanced RCC sensitivity to sunitinib [25].

The mammalian (mechanistic) target of rapamycin (mTOR) represents another key metabolic signaling pathway influencing a cellular tumorigenic phenotype by controlling vital processes, such as glucose and lipid metabolism, cell cycle, proliferation, metastasis, and chemo-resistance [26]. Significantly, enhanced activation of mTOR signaling has been reported in ovarian cancer [27, 28]. According to our results, combining MTF and SIM effectively targets the mTOR pathway by acting on PIK3R1. Also known as p85α, PIK3R1 serves as the regulatory subunit of PI3K. This subunit mediates the activation of the conventional PI3K/AKT signaling pathway, playing a significant role in tumorigenic processes such as cell growth, proliferation, metabolism, and angiogenesis [29]. Our results show that MTF and SIM synergistically decreased the expression levels of PIK3R1 in OVCAR-3 cells which was also validated on SKOV-3 cells. According to KEGG analysis, PI3K is activated by insulin receptor substrate 1 (IRS1), a signaling protein that is phosphorylated and activated upon the interaction between insulin growth factor and its receptor. According to KEGG, it can also be activated by stem cell factor (c-kit), SCF. C-kit is a typical proto-oncogene encoding a receptor tyrosine kinase [30], which interestingly, was also shown to be downregulated by combining MTF and SIM. Combining MTF and SIM targeted the main branch of PI3K, along with its activator, which in turn would possibly halt the tumorigenic effects of mTOR activation.

Our findings also indicated that combining MTF and SIM synergistically suppressed S-phase kinase-associated protein 2 (skp2) in ovarian cancer cells. Skp2, known to be upregulated in several types of cancers including ovarian cancer, possesses oncogenic activity due to its involvement in protein ubiquitination and degradation, subsequently regulating cellular metabolism, cell cycle and tumorigenesis [31]. SKP2 plays a pivotal role in controlling both negative and positive feedback loops to sustain the activity of the mTOR pathway. SKP2 tags its substrates through several types of ubiquitination including K63 and K48. Notably, AKT, a key component of the mTOR pathway is one of the K63 targets of skp2, through which it gets activated providing further skp2 reinforcement and creating a positive feedback mechanism. Additionally, SKP2 participates in the negative feedback mechanism of mTOR by mediating the degradation of RagA/B, RagC/D. Furthermore, SKP2 targets FOXO3 and FOXO1 by K48 ubiquitination. Therefore, with an overexpression of skp2, the proteolysis of FOXOs increases ultimately inhibiting AMPK. This dual action of inhibiting AMPK while sustaining mTOR stimulation positions SKP2 as a critical metabolic component in cellular regulation [32, 33]. Furthermore, another key tumor suppressor downregulated by skp2 is the negative cell cycle regulator p27 encoded by CDKN1B which was also shown in our study to be upregulated by the combination [34]. A meta-analysis by Lu et al. [35] showed that the loss of p27 is correlated with a worse outcome in ovarian cancer. Important to note that p53 indirectly suppresses cyclin/cdk complexes, increasing p27 levels [36]. The elevation of p27 in the presence of mutated p53 underscores its potential significance in apoptosis and cell cycle regulation, particularly in our cell lines where p53 function is impaired. An *in vitro* study by Xue et al. in triple-negative breast cancer cell lines showed that metformin synergistically with an insulin/IGF-1 receptor inhibitor suppressed skp2, which in turn stabilized p27. This axis is suggested to be one of the mechanisms responsible for the inhibition of cellular proliferation [37]. Consistent with this, a study by Wang et al. [38] also showed that simvastatin monotherapy increased p21 and p27 by decreasing skp2 leading to the activation of AMPK in hepatocellular carcinoma. In a study by Mudan et al. [31] Skp2 expression was shown to be significantly correlated with advanced clinical stages of ovarian carcinomas. The results suggest the role skp2 might play in OC progression. Results from this study were in conformity with a study by Shigemasa et al. [39] which showed that higher skp2 expression was observed in late-stage ovarian adenocarcinomas when compared with early disease stages. Overexpression of skp2 also showed a significant correlation with older age, poor patient survival, and high histological grades of the tumor. These results suggest that overexpression of SKP2 is a prognostic marker in patients with ovarian adenocarcinoma. These data suggest the potential benefit of suppressing skp2.

We also found that the combination of MTF and SIM synergistically suppressed the expression of ATP6V1D in ovarian cancer cells. ATP6V1D encodes a vacuolar ATPase (V-ATPase) component, a large primary rotary multi-subunit proton pump driven by ATP. mTORC1 is one of the targets of V-ATPase [40]. Indeed, a study by Marino et al. [41] showed that mTORC1 signaling is suppressed upon the inhibition of V-ATPase in Drosophila S2 cells.

Our study also found increased levels of TNFα expression when MTF and SIM were combined. Tumor necrosis factor α (TNFα) is a member of the cytokine family. After receptor activation, TNF signaling initiates apoptosis or induces cell survival and proliferation. The function of TNF is initially affected by the type of TNF receptor that is involved and physiological context.

Interestingly, our results also showed an increased expression of RhoA (RAS homolog) in OVCAR-3 but a decreased expression in SK-OV-3. Recent data from Kamel et al. [42] showed that simvastatin disrupts RhoA function. Consistent with our findings, the study demonstrated the activation of AMPK in osteosarcoma cells by simvastatin, with a further enhanced effect observed upon adding metformin to the regimen. RhoA belongs to the small GTPases family that are involved in various physiological processes such as cell morphology, and polarity [43]. Rho GTPases can be triggered and launch a cascade of signals via a variety of targets, comprising kinases and scaffold/adaptor-like proteins ultimately taking part in cellular survival, and proliferation. Indeed, overexpression of several members of Rho family of GTPases have been seen in several types of cancers including ovarian [43]. Horiuchi et al. showed that RhoA and RhoC were upregulated in advanced stage tumors particularly the serous histology as compared to initial staged [44]. Therefore, the variation of RhoA expression observed in our study warrants further analysis. We acknowledge the potential benefit of incorporating genome sequencing data for cells treated with standard chemotherapeutic drugs in combination with repurposed agents in this study.

## Conclusion and future perspectives

The objective of this study was to explore the molecular effects resulting from the combination of metformin (MTF) and simvastatin (SIM) in ovarian cancer cells. Our findings strongly indicate that the synergistic application of MTF and SIM can significantly reduce the viability and proliferation of ovarian cancer cells by targeting their metabolic activity through critical components within the metabolic signaling pathways network. Transcriptome profiling, conducted for the first time in this study, revealed a notable association with components of the metabolic sensors, namely AMPK and mTOR. Through transcriptome profiling, this study identified differentially expressed genes worth investigating. These DEGs correspond to a broad network of signaling pathways that could serve as targeted interventions in Ovarian Cancer.

## Supporting information

S1 Fig **A–AMPK signaling pathway**: AMP-activated protein kinase (AMPK) is a serine threonine kinase that is highly conserved through evolution. AMPK system acts as a sensor of cellular energy status. It is activated by increases in the cellular AMP:ATP ratio caused by metabolic stresses that either interfere with ATP production (eg, deprivation for glucose or oxygen) or that accelerate ATP consumption (eg, muscle contraction). Several upstream kinases, including liver kinase B1 (LKB1), calcium/calmodulin kinase kinase-beta (CaMKK beta), and TGF-beta-activated kinase-1 (TAK-1), can activate AMPK by phosphorylating a threonine residue on its catalytic alpha-subunit. Once activated, AMPK leads to a concomitant inhibition of energy-consuming biosynthetic pathways, such as protein, fatty acid and glycogen synthesis, and activation of ATP-producing catabolic pathways. **B–mTOR signaling pathway**: The mammalian (mechanistic) target of rapamycin (mTOR) is a highly conserved serine/threonine protein kinase, which exists in two complexes termed mTOR complex 1 (mTORC1) and 2 (mTORC2). mTORC1 contains mTOR, Raptor, PRAS40, Deptor, mLST8, Tel2 and Tti1. mTORC1 is activated by the presence of growth factors, amino acids, energy status, stress and oxygen levels to regulate several biological processes, including lipid metabolism, autophagy, protein synthesis and ribosome biogenesis. On the other hand, mTORC2, which consists of mTOR, mSin1, Rictor, Protor, Deptor, mLST8, Tel2 and Tti1, responds to growth factors and controls cytoskeletal organization, metabolism and survival.
(ZIP)

S1 Table Primer sequences. Table showing the forward and reverse sequences of the primers for the chosen differentially expressed genes used for real-time PCR. Primers were designed using Primer-BLAST designing tool.
(PDF)

S2 Table DEGs combination vs control group. Table showing the full list of the DEGs found in the combination vs control group in an ascending order of p-values. Columns show the following: Probe ID (array ID), gene ID, gene symbol, fold change for each group (with insignificant values for single therapies), p-values with adjusted p-values.
(XLSX)
