# Supplementary figures and images for: Evaluating synergistic effects of metformin and simvastatin on ovarian cancer cells
Source: PLoS One. 2024 Mar 15;19(3):e0298127. doi: 10.1371/journal.pone.0298127 (PMC10942021; doi:10.1371/journal.pone.0298127)

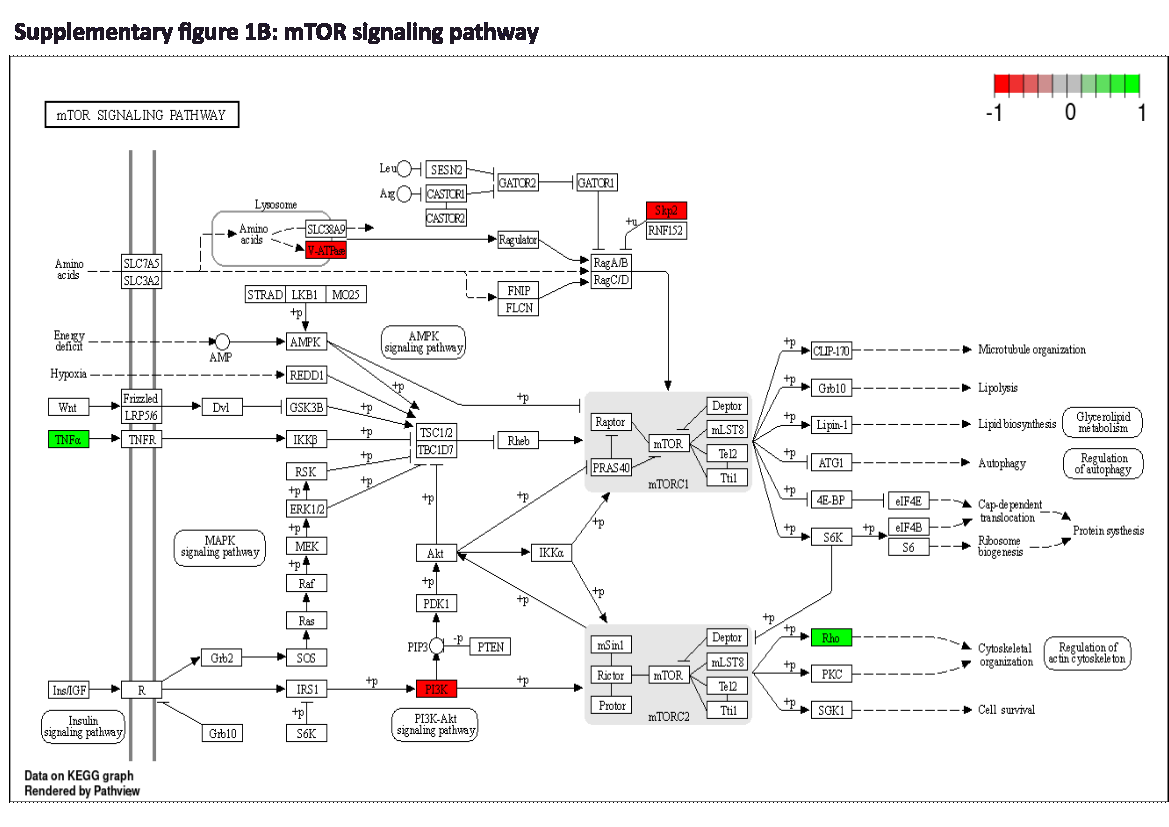

Supplement: S1 Fig — A–AMPK signaling pathway: AMP-activated protein kinase (AMPK) is a serine threonine kinase that is highly conserved through evolution. AMPK system acts as a sensor of cellular energy status. It is activated by increases in the cellular AMP:ATP ratio caused by metabolic stresses that either interfere with ATP production (eg, deprivation for glucose or oxygen) or that accelerate ATP consumption (eg, muscle contraction). Several upstream kinases, including liver kinase B1 (LKB1), calcium/calmodulin kinase kinase-beta (CaMKK beta), and TGF-beta-activated kinase-1 (TAK-1), can activate AMPK by phosphorylating a threonine residue on its catalytic alpha-subunit. Once activated, AMPK leads to a concomitant inhibition of energy-consuming biosynthetic pathways, such as protein, fatty acid and glycogen synthesis, and activation of ATP-producing catabolic pathways. B–mTOR signaling pathway: The mammalian (mechanistic) target of rapamycin (mTOR) is a highly conserved serine/threonine protein kinase, which exists in two complexes termed mTOR complex 1 (mTORC1) and 2 (mTORC2). mTORC1 contains mTOR, Raptor, PRAS40, Deptor, mLST8, Tel2 and Tti1. mTORC1 is activated by the presence of growth factors, amino acids, energy status, stress and oxygen levels to regulate several biological processes, including lipid metabolism, autophagy, protein synthesis and ribosome biogenesis. On the other hand, mTORC2, which consists of mTOR, mSin1, Rictor, Protor, Deptor, mLST8, Tel2 and Tti1, responds to growth factors and controls cytoskeletal organization, metabolism and survival. (ZIP) [file pone.0298127.s001.zip › supplementary fig 1B. mTOR.tif]

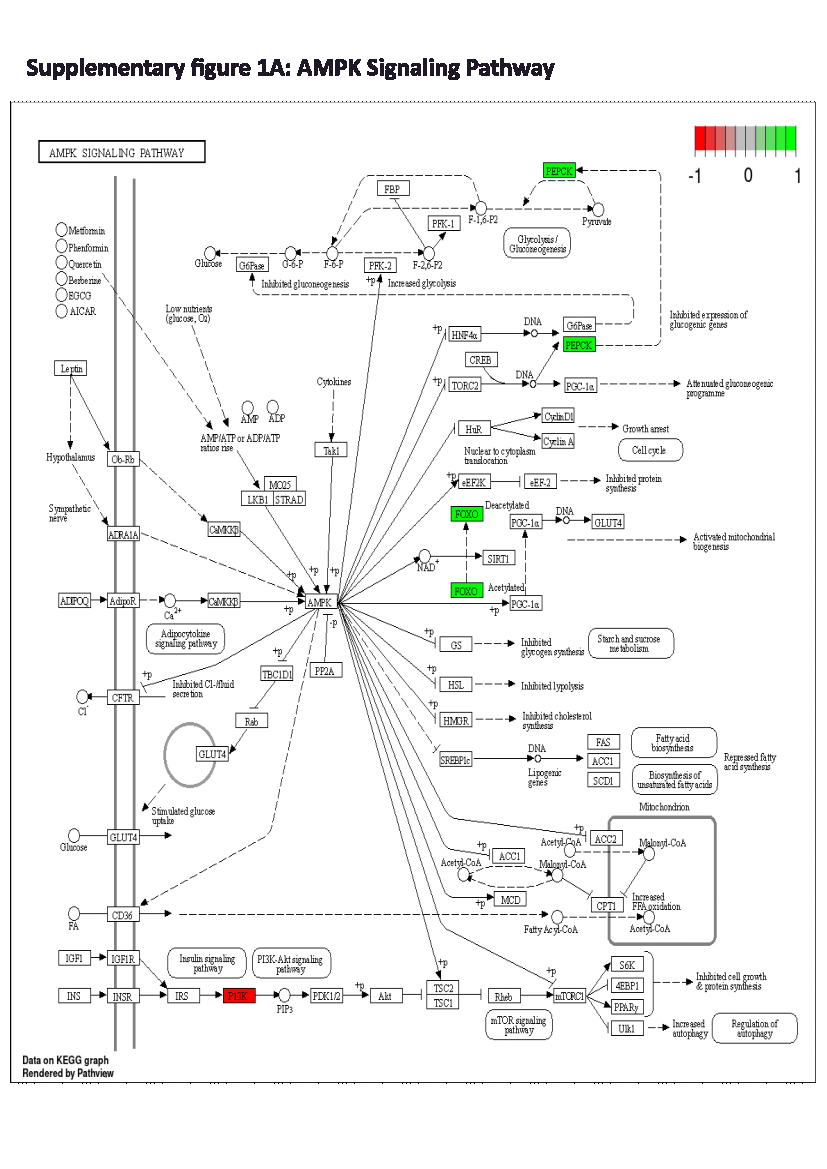

Supplement: S1 Fig — A–AMPK signaling pathway: AMP-activated protein kinase (AMPK) is a serine threonine kinase that is highly conserved through evolution. AMPK system acts as a sensor of cellular energy status. It is activated by increases in the cellular AMP:ATP ratio caused by metabolic stresses that either interfere with ATP production (eg, deprivation for glucose or oxygen) or that accelerate ATP consumption (eg, muscle contraction). Several upstream kinases, including liver kinase B1 (LKB1), calcium/calmodulin kinase kinase-beta (CaMKK beta), and TGF-beta-activated kinase-1 (TAK-1), can activate AMPK by phosphorylating a threonine residue on its catalytic alpha-subunit. Once activated, AMPK leads to a concomitant inhibition of energy-consuming biosynthetic pathways, such as protein, fatty acid and glycogen synthesis, and activation of ATP-producing catabolic pathways. B–mTOR signaling pathway: The mammalian (mechanistic) target of rapamycin (mTOR) is a highly conserved serine/threonine protein kinase, which exists in two complexes termed mTOR complex 1 (mTORC1) and 2 (mTORC2). mTORC1 contains mTOR, Raptor, PRAS40, Deptor, mLST8, Tel2 and Tti1. mTORC1 is activated by the presence of growth factors, amino acids, energy status, stress and oxygen levels to regulate several biological processes, including lipid metabolism, autophagy, protein synthesis and ribosome biogenesis. On the other hand, mTORC2, which consists of mTOR, mSin1, Rictor, Protor, Deptor, mLST8, Tel2 and Tti1, responds to growth factors and controls cytoskeletal organization, metabolism and survival. (ZIP) [file pone.0298127.s001.zip › supplementary figure 1A. AMPK.tif]
