# Supplementary material for: Evaluating synergistic effects of metformin and simvastatin on ovarian cancer cells
Source: PLoS One. 2024 Mar 15;19(3):e0298127. doi: 10.1371/journal.pone.0298127 (PMC10942021; doi:10.1371/journal.pone.0298127)
Supplement: S1 Table — Table showing the forward and reverse sequences of the primers for the chosen differentially expressed genes used for real-time PCR. Primers were designed using Primer-BLAST designing tool. (PDF) [file pone.0298127.s002.pdf]

Supplementary Table 1: Primer sequences

Primer		
**GAPDH**	Forward	GGAGTCCACTGGCGTCTTCAC
	Reverse	GAGGCATTGCTGATGATCTTGAGG
**FOXO3**	forward	TCTACGAGTGGATGGTGCGTT
	reverse	CGACTATGCAGTGACAGGTTGT
**PCK2**	Forward	AAACCTGGTATGTGCGGTGG
	Reverse	AGATGGTTGGAGGTTGTGGAG
**PIK3R1**	Forward	ATGTTAGCTTTGGGAAGGGG
	Reverse	CGTACCAAAAAGGTCCCGTC
**SKP2**	Forward	ACTTTCCAGGTCTGATGAGTCT
	Reverse	TCACCCCTTGAGACAGCAAC
**RhoA**	Forward	AAGCAGGTAGAGTTGGCTTTGTG
	Reverse	ATCGGTATCTGGGTAGGAGAGG
**ATP6V1D**	Forward	AAGCCAAGTTCACAGCAGGT
	Reverse	CGCTCGAATCTTCACTTGCG
**TNF**	Forward	CACAGTGAAGTGCTGGCAAC
	Reverse	GATCAAAGCTGTAGGCCCCA
**SEMA7A**	Forward	CTGTTTGAAGGGGACGAGGTGT
	Reverse	ACGATGGTGGCTTTGATGAACTG
**PHACTR2**	Forward	GGGTAGAAGGTGAGGGGACC
	Reverse	GGCTCCTTTGGTCCTTCGAT
